# Health-related quality of life in patients with metastatic renal cell carcinoma treated with sunitinib *vs* interferon-*α* in a phase III trial: final results and geographical analysis

**DOI:** 10.1038/sj.bjc.6605552

**Published:** 2010-01-26

**Authors:** D Cella, M D Michaelson, A G Bushmakin, J C Cappelleri, C Charbonneau, S T Kim, J Z Li, R J Motzer

**Affiliations:** 1 Robert H Lurie Comprehensive Cancer Center of Northwestern University, 710 N Lake Shore Dr., Chicago, IL 60611, USA; 2 Massachusetts General Hospital, Cancer Center, 55 Fruit Street, YAW 7, Boston, MA 02114, USA; 3 Pfizer Global Research and Development, 50 Pequot Avenue, New London, CT 06320, USA; 4 Pfizer Global Research and Development, 235 42nd Street, New York, NY 10017, USA; 5 Pfizer Oncology, La Jolla, 11095 Torreyana Road, San Diego, CA 92121, USA; 6 Memorial Sloan-Kettering Cancer Center, 1275 York Avenue, New York, NY 10021, USA

**Keywords:** health-related quality of life, metastatic renal cell carcinoma, patient-reported outcomes, receptor tyrosine kinase inhibitor, sunitinib

## Abstract

**Background:**

In a randomised phase III trial, sunitinib significantly improved efficacy over interferon-*α* (IFN-*α*) as first-line therapy for metastatic renal cell carcinoma (mRCC). We report the final health-related quality of life (HRQoL) results.

**Methods:**

Patients (*n*=750) received oral sunitinib 50 mg per day in 6-week cycles (4 weeks on, 2 weeks off treatment) or subcutaneous IFN-*α* 9 million units three times weekly. Health-related quality of life was assessed with nine end points: the Functional Assessment of Cancer Therapy–General and its four subscales, FACT–Kidney Symptom Index (FKSI-15) and its Disease-Related Symptoms subscale (FKSI-DRS), and EQ-5D questionnaire's EQ-5D Index and visual analogue scale. Data were analysed using mixed-effects model (MM), supplemented with pattern-mixture models (PMM), for the total sample and the US and European Union (EU) subgroups.

**Results:**

Patients receiving sunitinib reported better scores in the primary end point, FKSI-DRS, across all patient populations (*P*<0.05), and in nine, five, and six end points in the total sample, in the US and EU groups respectively (*P*<0.05). There were no significant differences between the US and EU groups for all end points with the exception of the FKSI item ‘I am bothered by side effects of treatment’ (*P*=0.02). In general, MM and PMM results were similar.

**Conclusion:**

Patients treated with sunitinib in this study had improved HRQoL, compared with patients treated with IFN-*α*. Treatment differences within the US cohort did not differ from those within the EU cohort.

In the United States and Europe, kidney cancer is estimated to account for approximately 4% of all new cases of cancer (Jemal *et al*, 2008). The annual incidence of cancer of the kidney and renal pelvis in the United States in 2008 is estimated to be 54 390, with approximately 13 010 deaths per year (Jemal *et al*, 2008). In the 25 countries making up the European Union (EU), more than 63 000 new cases of renal cell carcinoma (RCC) were diagnosed in 2006, resulting in an estimated 26 000 deaths annually (Ferlay *et al*, 2007).

The prognosis for patients with metastatic RCC (mRCC) has been historically poor, with only 10% of patients surviving beyond 5 years (US National Institutes of Health, 2006). Patients living with mRCC can suffer significant symptoms, even with the resection of primary tumours and metastatic sites (Williams *et al*, 2004). Health-related quality of life (HRQoL) outcomes are an important assessment tool to measure disease- and treatment-related symptoms directly reported by patients.

A number of health status scales are available for the measurement of HRQoL and disease- and treatment-related symptoms in cancer patients (de Haes *et al*, 1990; Cella *et al*, 1993; Rabin and de Charro, 2001), including two validated scales that are specific to RCC: the Functional Assessment of Cancer Therapy–Kidney Symptom Index (FKSI) and the RCC Symptom Index (Cella *et al*, 2006; Harding *et al*, 2007). Factors such as age, race, marital status, education level, income, and employment have been shown to affect HRQoL in cancer patients (Movsas *et al*, 2006) and may influence outcomes assessed using these scales. Cultural differences may also affect patients’ responses to questionnaires (Wild *et al*, 2005). These variables should be considered when interpreting data and making regional comparisons from international multi-centre studies.

Sunitinib malate is an oral multi-targeted receptor tyrosine kinase inhibitor of vascular endothelial growth factor receptors 1, 2, and 3, platelet-derived growth factor receptors, stem cell factor receptor, colony-stimulating factor 1 receptor, glial cell-line-derived neurotrophic factor receptor (Rearranged during Transfection), and FMS-like tyrosine kinase 3, with anti-tumour and anti-angiogenic effects (Abrams *et al*, 2003; Mendel *et al*, 2003; Murray *et al*, 2003; O’Farrell *et al*, 2003; Kim *et al*, 2006; Pfizer Ltd, 2009). Sunitinib has been approved multinationally for the treatment of advanced RCC and for gastrointestinal stromal tumours after disease progression on or intolerance to imatinib mesylate therapy (Pfizer Ltd, 2009).

In an international randomised phase III trial, sunitinib showed statistically significant improvement in progression-free survival and objective response rate compared with interferon-*α* (IFN-*α*) as first-line therapy for mRCC (*P*<0.001), and longer median overall survival compared with IFN-*α* (Motzer *et al*, 2007, 2009). An interim analysis of the trial data also showed superior HRQoL benefits with sunitinib compared with IFN-*α* (Motzer *et al*, 2007; Cella *et al*, 2008) . Here we report updated HRQoL results based on the final data for this trial, including an analysis of geographical differences.

## Methods

Full details of the study design have been previously reported by Motzer *et al* (2007). This study was conducted in accordance with the Declaration of Helsinki Principles and Good Clinical Practice Guidelines. All patients provided written informed consent.

### Patients and study design

Male and female patients aged ⩾18 years with mRCC with a component of clear cell histology were eligible for enrollment in this study. Other eligibility criteria have been previously reported (Motzer *et al*, 2007). Patients were excluded if they had a severe, acute, or chronic medical or psychiatric condition, or a laboratory abnormality that could increase the risk associated with study participation or study drug administration, or that could interfere with the interpretation of study results, or in the judgment of the investigator makes the patient inappropriate for entry.

In this phase III trial, patients were randomised to receive either sunitinib or IFN-*α* in repeated 6-week cycles: oral sunitinib was initiated at a starting dose of 50 mg per day on a schedule of 4 weeks on treatment followed by 2 weeks off treatment (Schedule 4/2) and IFN-*α* was administered as a subcutaneous injection on 3 non-consecutive days per week at a dose of three million units (MU) in the first week, six million units in the second week, and nine million units thereafter. Dose modifications were allowed for toxicity management in both treatments.

### Health-related quality of life assessments

Health-related quality of life was assessed using three validated self-reported questionnaires: the FKSI-15 (Cella *et al*, 2006), the Functional Assessment of Cancer Therapy–General (FACT-G) (Cella *et al*, 1993), and EuroQoL Group's EQ-5D self-report questionnaire (Rabin and de Charro, 2001). Full details of these questionnaires and their application in this study were reported by Cella *et al* (2008).

The questionnaires were completed on days 1 and 28 of each 42-day treatment cycle, and at the end of treatment or on study withdrawal. Non-English speakers were provided with questionnaires in their preferred language.

Nine HRQoL end points were derived from the three questionnaires, including: (1) the FKSI-15 total score, (2) FKSI-15's FKSI-Disease-Related Symptoms (FKSI-DRS) subscale (Cella *et al*, 2007), and (3) the FACT-G total score, (4–7) FACT-G's four subscales: physical, social/family, emotional, and functional well-being (PWB, SFWB, EWB, and FWB respectively), and (8–9) EQ-5D questionnaire's EQ-5D Index (EuroQol, 1990; Rabin and de Charro, 2001) and visual analogue scale (EQ-VAS) (de Boer *et al*, 2004). The FKSI-DRS subscale score was prospectively specified as the primary HRQoL end point.

All HRQoL end points were reported for the total sample (US, EU, Australia, Brazil, Canada, and Russia). In addition, these end points were examined to determine any differences between treatment arms and between the US and European (EU; France, Germany, Italy, Poland, Spain, United Kingdom) groups.

### Statistical analyses

All analyses were conducted on the intention-to-treat population. Patient demographics and characteristics were described using frequency distributions, means, and standard deviations. Completion of the questionnaires was defined as responses to more than 80% of items in the overall FACT-G and more than 50% of items in the FKSI-15, FKSI-DRS, and FACT-G subscales. All patients with a baseline assessment and at least one post-baseline measurement were included in the analysis. Patients (*n*=25) who crossed over from IFN-*α* to sunitinib treatment were included in the analyses with the original randomisation assignment. Patients with no post-baseline assessment were excluded.

Estimated (or predicted) means were calculated for each end point and for each treatment, as estimated using the repeated-measures mixed-effects model (MM), controlling for time, treatment, country, treatment-by-time, and treatment-by-country interactions, and baseline (cycle 1, day 1) score (Fairclough, 2002; Singer and Willett, 2003; Fitzmaurice *et al*, 2004). Means within treatment group and differences in means between treatment groups were estimated across the entire span of the post-baseline period and all available observations.

With the exception of the individual items of FKSI-15, sensitivity analyses to MM on HRQoL total and subscale scores were performed using pattern-mixture models (PMM) (Little, 1994; Hedeker and Gibbons, 1997), which helps interpret results when data are not missing at random. Results are not overly dependant on the nature of missing data if results from MM and PMM are similar. The main difference from MM is the addition of the new variable ‘Pattern’ and interaction terms of ‘Pattern’ variable with all other predictors (except ‘Baseline’). Patterns were defined according to the dynamics of the attrition process.

In applying the pattern-mixture methodology, we needed to choose the number of patterns and how they are distributed over the study population. We graphed the percentage of patients with data up to a certain cycle. From this depiction three distinct patterns emerged: an exponential decrease in the number of patients from cycle 1 to cycle 10, then a modest linear decrease in the number of patients from cycle 11 to cycle 21; followed by a more pronounced linear decrease in the number of patients after cycle 21. On the basis of these observations, we selected three patterns with each cycle belonging to one of the patterns.

Data were analysed using SAS 8.2 (SAS Institute, Cary, NC, USA). Statistical significance for between-treatment differences was set at *P*<0.05. No adjustments were made for multiple comparisons in this supplemental analysis.

Estimated means for US and EU groups were calculated for each end point and each treatment arm based on the model described above. Estimations for the EU group were performed over the balanced population, that is, as if every country comprising the EU group had the same number of patients in the study.

## Results

### Patient baseline characteristics

A total of 750 patients were randomly assigned to receive sunitinib (*n*=375) or IFN-*α* (*n*=375). The US group consisted of 347 patients. The EU group (274 patients) comprised patients enrolled in France (*n*=82), Germany (*n*=17), Italy (*n*=24), Poland (*n*=103), Spain (*n*=27), and the UK (*n* =21). Patients were evenly distributed between the two treatments arms within each geographical group and there were no significant differences between treatment groups (Table 1). Patients had received up to 30 cycles of treatment at the time of the final analysis.

### Questionnaire completion rates

A total of 692 patients (92%) had at least one post-baseline observation for each of the FACT-G, FKSI-15, and EQ-5D questionnaires. In the US group, an equal number of patients, 320 (92%) each, completed the FACT-G and FKSI-15 questionnaires for at least one treatment cycle; 319 patients (92%) completed the EQ-5D questionnaire. In the EU group 252 (92%), 252 (92%), and 253 (92%) patients completed the FACT-G, FKSI-15, and EQ-5D questionnaires respectively.

In both study populations, completion rates were slightly lower in the IFN-*α* treatment arm compared with the sunitinib treatment arm. The completion rates in the US sample were 96.6% (173 of 179) for sunitinib and 87.5% (147 of 168) for IFN-*α*; the completion rates in the EU sample were 96.3% (130 of 135) for sunitinib and 87.8% (122 of 139) for IFN-*α*.

### Questionnaire assessments

#### Primary HRQoL end point: FKSI-DRS

For the primary end point, FKSI-DRS, differences in estimated means significantly favoured sunitinib over IFN-*α* in the total sample and in both the US and EU groups (all *P*'s<0.05; Table 2).

Patients receiving sunitinib reported higher FKSI-DRS scores than those receiving IFN-*α*, with a significant difference in the overall means (2.36, *P*<0.0001; MM; Table 2). In examining the nine items in the FKSI-DRS (Table 3, bold items), the differences in means significantly favoured sunitinib (*P*<0.05) in six of nine items (lack of energy, fatigue, coughing, breathlessness, weight loss, and fever).

### Secondary HRQoL end points

As with the primary end point (FKSI-DRS), differences in estimated means for FKSI-15 (total score), FACT-G (total score and all domains), EQ-5D Index, and EQ-VAS were all significantly favourable for sunitinib compared with IFN-*α* in the total sample (all *P*'s<0.05; Table 2).

In the US group, all end points, with the exception of the EQ-5D scores, were significantly better in the sunitinib group than in the IFN-*α*. In the EU group, between-treatment differences were significant in five of nine end points favouring sunitinib over IFN-*α* (Table 2). There were no significant treatment differences between the US and EU groups for all of these total and subscale scores for the HRQoL end points (Table 2).

#### FKSI-15

Higher (more favourable) FKSI-15 scores at each cycle were observed for sunitinib treatment than for IFN-*α* in patients in the total sample (Tables 2 and 3). Patients on sunitinib treatment reported higher FKSI-15 scores than those on IFN-*α* treatment with a significant difference in the overall means (4.06, *P*<0.0001; MM, Table 2). The difference in means significantly favoured sunitinib compared to IFN-*α* (*P*<0.05) for 10 of the 15 FKSI items in the total sample (Table 3). Interferon-*α* was not superior to sunitinib in any of the items in the subscales. Between-treatment differences did not significantly differ between US and EU groups across all end points, with the exception of FKSI symptom ‘I am bothered by side effects of treatment’ (*P*=0.0209; Table 3).

#### FACT-G assessments

The differences in scores between patients receiving sunitinib and those receiving IFN-*α* were statistically significant for the FACT-G total score in the total sample and in both US and EU groups (Table 2). Patients receiving sunitinib reported higher FACT-G scores than those receiving IFN-*α*, with a significant difference in the overall means (6.62, *P*<0.0001; 6.08, *P*<0.0001; 4.83, *P*=0.0036 respectively; MM).

Similarly, for the FACT-G subscales, differences in scores between the two treatment groups significantly favoured sunitinib over IFN-*α* in the total sample and in the US groups. In the EU group the differences in score between the two treatments were not significant for three of the four subscales (Table 2).

#### EuroQoL assessments

The overall post-baseline mean treatment difference for the EQ-5D Index in the total sample was estimated to be 0.05 points in favour of sunitinib (*P*=0.0078; Table 2). The overall mean treatment difference for EQ-VAS was estimated to be 7.70 in favour of sunitinib (*P*<0.0001; Table 2).

In the US and EU groups, the differences between the two treatment groups were not significant for EQ-5D score, but were significant for EQ-VAS score (*P*=0.0076 and 0.0177 respectively).

### Sensitivity analyses

**Figure 1:**
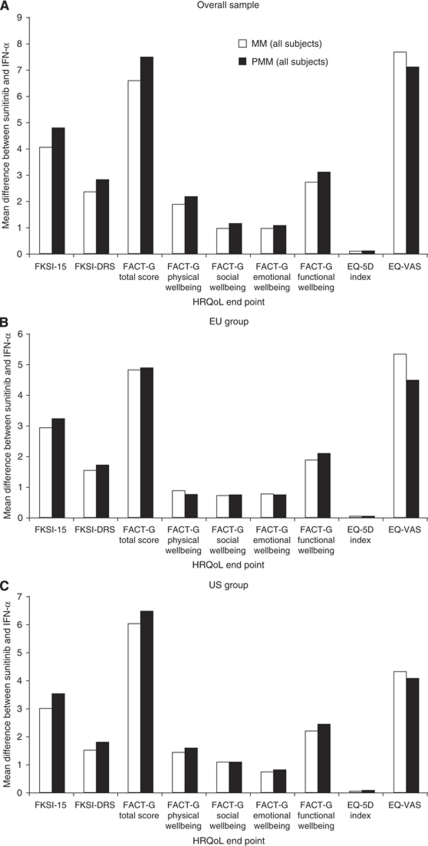
(**A**–**C**) Comparison of results between MM and PMM analyses across all available post-baseline observations. MM, mixed-effects model difference between sunitinib and IFN-*α*; PMM, pattern-mixture model difference between sunitinib and IFN-*α*; FKSI-15, FACT–Kidney Symptom Index-15 item; FKSI-DRS, FKSI Disease-Related Symptoms subscale; FACT-G, Functional Assessment of Cancer Therapy–General; EQ-5D Index, EuroQoL health-utility index; EQ-VAS, EQ visual analogue scale.

In general, the PMM results were consistent with those results from the MM (Figure 1). Similar to the MM results on the total and subscale scores, the MM results on the total and subscale scores of HRQoL did not show any statistical discrepancy between US treatment differences and EU treatment differences (Figure 1).

## Discussion

In this phase III trial, sunitinib was associated with superior HRQoL compared with IFN-*α* in patients with mRCC, *P*<0.01 as measured by overall health status (EQ-5D Index and EQ-VAS), cancer-specific HRQoL (FACT-G and its subscales), and *P*<0.0001 as measured by kidney cancer-related symptoms (FKSI-15 and FKSI-DRS) (Cella *et al*, 2008). The results reflect between-treatment differences rather than within-treatment improvement compared with baseline.

Although patients were aware of the assigned treatment arm, which could potentially bias the responses to the HRQoL questionnaires in favour of sunitinib, this was substantially mitigated by several factors. Assessments were conducted and measured uniformly between treatment groups, and through control of the baseline HRQoL covariates and use of the random-effects model, the analysis incorporated and controlled for the propensity to respond in a certain way.

Overall, FKSI-DRS scores, the primary HRQoL end point of this study, showed that patients receiving sunitinib had fewer severe disease-specific symptoms (lack of energy, fatigue, coughing, breathlessness, weight loss, and fever) than did patients treated with IFN-*α*. Patients receiving sunitinib also reported better scores for FKSI-15, FACT-G, EQ-5D Index, and EQ-VAS, the secondary HRQoL end points. Although it cannot be ruled out, it is unlikely that these post-treatment differences could have been due to unobserved pre-treatment differences in comorbid conditions (or other factors) as the large number of patients randomised to each treatment would be expected to make the treatment groups equivalent in known and unknown ways. Therefore, any noticeable post-treatment difference is most reasonably attributable to the intervention, which was controlled by random assignment. Moreover, if baseline comorbid conditions (or other factors) were related to the HRQoL outcome score, they would also likely be related to their corresponding HRQoL baseline score, which was adjusted for in the model (thereby increasing the precision of estimated treatment effects).

No significant differences were found between the US and EU groups for the FKSI-DRS, FACT-G, EQ-5D, and EQ-VAS, implying no difference in treatments (sunitinib *vs* IFN-*α*) on HRQoL outcomes in the US and EU subpopulations. FKSI-15 symptoms also did not differ significantly between the US and EU subgroups (with the exception of ‘I am bothered by side effects of treatment’).

For the item ‘I am bothered by side effects of treatment’, the geographical variation observed between the US and EU subgroups may reflect many factors, including a chance variation, a genuine pharmacogenetic variation, cultural differences in attitudes to illness, differences in health-care delivery or patients’ experiences, or differences in scoring and reporting of HRQoL outcomes. But, more likely, the geographical variation observed between the US and EU subgroups is a trivial anomaly because the treatment difference within each subgroup was not statistically significant (*P*>0.05).

Several interesting observations are worthy of comment. The subscales of the FACT-G (physical, social, and emotional well-being subscales) and items within the FKSI-15 were significantly different by treatment group in the US but not in the EU subgroups (i.e., ‘I feel fatigued’, ‘I have been coughing’, ‘I am able to enjoy life’, ‘I worry that my condition will get worse’). In addition, the FKSI question ‘I am bothered by side effects of treatment’ was not significantly different in the patients in either the IFN-*α* or the sunitinib arm, either when analysed in the entire group or when analysed in the US or EU subgroups. Further, only 4 items within the 15-item FKSI significantly differentiated treatment groups in both the US and the EU populations. These four items were ‘I have a lack of energy,’ ‘I have been short of breath,’ ‘I am able to work,’ and ‘I am bothered by fevers.’ The other items on the FKSI scale did not seem to be important in distinguishing HRQoL in the IFN-*α* and sunitinib treatment arms in both the US and the EU treatment groups.

The EU group may give more variability in responses than the US group. This would not necessarily imply any cross-cultural issues, especially as the translated questions were validated in European patients. More research would inform us further, including psychometric testing of the FKSI in diverse European samples as the instrument was developed and validated only in English-speaking patients in the United States.

Individual items have more random variability (measurement error) than multiple-item subscales, which tend to be more reliable and accurate (Sloan *et al*, 2002). Therefore, it is not surprising that several items on the FKSI scale did not distinguish HRQoL in the IFN-*α* and sunitinib treatment arms in both the US and the EU groups. What is important, though, is the direction of the effect: the estimated treatment effect was in the same direction in the two geographical groups for 14 of 15 FKSI items.

It should be noted that the primary HRQoL end point, FKSI-DRS, showed treatment differences within the US group and the EU group, but not between these two groups (*P*=0.9645). The other end points are considered secondary outcomes in this study.

The main purpose of the geographical analysis was to determine whether the treatment effect within the US group differs from the treatment effect within the EU group. Despite there being a significant effect (*P*<0.05) for some subscales and items in the US group but not in the EU group, the treatment effect within the US group did not really differ from that within the EU group. Such occurrences are not uncommon. For example, an active intervention group may show a significant change from baseline but the control group may not. If there is no difference in the mean changes between the two treatment groups, we would conclude that there is no treatment effect between the two treatment groups.

The MM used in these analyses reduced the potential for bias caused by the varying numbers of patients in the two treatments leaving the study over a period of time as a result of differing efficacy. The results from the PMM, which allowed comparison between different treatments based on the pattern of missing data, supported and validated these findings. These results, therefore, showed the robustness of the data from this analysis showing that sunitinib was effective across all patient populations irrespective of country, cultural, and treatment differences.

All patients including those who crossed over from IFN-*α* to sunitinib treatment were analysed as per original randomisation assignment. The impact of such analyses, if anything, can be expected to make results of sunitinib benefit more conservative. Yet, even with the inclusion of these 25 crossover patients, sunitinib showed HRQoL benefits over IFN-*α*.

A recent geographical analysis of interim data from this study, which included a European-only sample and data from only six treatment cycles, reported similar results and conclusions overall (Castellano *et al*, 2009). Some variations in results between these two analyses probably stem from their use of different models, different sets of data, and different objectives and hypotheses.

The results from our final analyses of HRQoL outcomes are consistent with the previously reported interim results from the overall sample (Cella *et al*, 2008) which reported superior HRQoL outcomes for sunitinib over IFN-*α*. In addition, the similarity in findings for patients in the geographical subsamples suggests that regional variations in treatment experience or underlying cultural differences in HRQoL reporting are minimal. Although some demographic variables were statistically significant between the EU and US groups, in general, the differences were caused by the relatively large sample sizes and did not have real import.

## Conclusions

In this study, patients treated with sunitinib had improved HRQoL compared with patients treated with IFN-*α*. In general, treatment differences within the US cohort did not differ from treatment differences within the EU cohort.

## Figures and Tables

**Table 1:** Baseline patient characteristics

	**Total sample (*n*=750)**		**US group (*n*=347)** a		**EU group (*n*=274)** a		**Total US *vs* total EU**
	**Sunitinib (*n*=375)**	**IFN-*α* (*n*=375)**	**Sunitinib (*n*=179)**	**IFN-*α* (*n*=168)**	**Sunitinib (*n*=135)**	**IFN-*α* (*n*=139)**	**diff. US *vs* EU *P*-value**
Mean age, years (range)	61 (27–87)	60 (34–85)	61 (39–87)	60 (34–85)	61 (27–80)	60 (39–82)	0.8735
Male/female (%)	71/29	72/28	78/22	65/35	65/35	83/17	0.5172
*Race/Ethnicity (%)*							0.0004 (<0.0001^b^)
Asian	1.87	3.20	1.68	3.57	0.74	0.72	
Black	1.07	2.67	1.12	4.76	0.74	1.44	
Not allowed to ask	0.27	0.27	0	0.60	0	0	
Not listed	2.40	3.47	5.03	5.95	0	1.44	
White	94.40	90.40	92.18	85.12	98.52	96.40	
Previous nephrectomy (%)	90	89	92	91	90	91	0.7363
Previous radiation therapy (%)	14	14	13	12	16	14	0.3428
							
*Common sites of metastases (%)*							
Lung	78	79	83	82	71	77	0.0152
Liver	26	24	30	27	24	19	0.0601
Bone	30	30	31	26	31	30	0.5115
Lymph nodes	58	53	53	46	67	60	0.0004
*Number of disease sites (%)*							0.0837
1	14	19	11	18	17	25	
2	29	30	31	30	27	28	
⩾3	57	51	58	51	56	47	
*ECOG PS (%)*							0.0115 (0.0075^c^)
0	62	61	67	68	57	58	
1	38	38	33	31	43	40	
2	0	1	0	1	0	2	
							

Abbreviations: IFN-*α*=interferon-*α*; ECOG PS=Eastern Cooperative Oncology Group.

a In our previous publication (Cella *et al*, 2008) one patient in the US sample was inadvertently assigned to the EU sample. This paper, with 274 (instead of 275) patients in the EU group and 347 (instead of 346) in the US group, gives the correct allocation.

b All but ‘white’ races collapsed in a ‘not white’ category.

c ECOG 1 and 2 collapsed in one category, ECOG ⩾1.

**Table 2:** Model means of HRQoL end points across all available post-baseline observations (mixed-effects model)

	**Total group**				**EU group**				**US group**				**US *vs* EU**	
**Symptoms and HRQoL end points**	**SU**	**IFN-*α***	**Diff.**	***P*-value**	**SU**	**IFN-*α***	**Diff.**	***P*-value**	**SU**	**IFN-*α***	**Diff.**	***P*-value**	**Diff. US *vs* diff. EU**	***P*-value**
FKSI-15	45.47	41.41	4.06	<0.0001^*^	44.91	41.99	2.93	0.0051^*^	45.40	42.36	3.04	<0.0001^*^	0.11	0.9203
FKSI-DRS	29.90	27.53	2.36	<0.0001^*^	29.53	27.99	1.54	0.0048^*^	29.29	27.72	1.57	<0.0001^*^	0.03	0.9645
FACT-G total score	80.49	73.88	6.62	<0.0001^*^	79.34	74.51	4.83	0.0036^*^	82.84	76.76	6.08	<0.0001^*^	1.25	0.4919
*Physical well-being*	21.61	19.72	1.89	0.0004^*^	21.30	20.43	0.87	0.2064	21.40	19.93	1.48	0.0023^*^	0.60	0.4353
*Social well-being*	22.46	21.51	0.95	0.0127^*^	21.78	21.08	0.71	0.1297	23.83	22.69	1.13	0.0044^*^	0.43	0.4099
*Emotional well-being*	18.17	17.18	0.98	0.0023^*^	17.92	17.14	0.77	0.0548	18.72	17.94	0.78	0.0175^*^	0.01	0.9807
*Functional well-being*	18.18	15.45	2.73	<0.0001^*^	17.77	15.88	1.89	0.0084^*^	19.47	17.23	2.25	<0.0001^*^	0.36	0.6477
EQ-5D Index	0.75	0.69	0.05	0.0078^*^	0.72	0.71	0.01	0.7127	0.77	0.75	0.02	0.2467	0.01	0.6862
EQ-VAS	73.95	66.25	7.70	<0.0001^*^	72.55	67.22	5.33	0.0177^*^	75.50	71.14	4.36	0.0076^*^	−0.97	0.6980

Abbreviations: SU=sunitinib; IFN-*α*=interferon-*α*; Diff.=sunitinib *vs* IFN-*α*; FKSI-15=FACT–Kidney Symptom Index15 item; FKSI-DRS=FKSI disease-related symptom subscale. FACT-G=Functional Assessment of Cancer Therapy–General; EQ-5D Index=EuroQoL health-utility index^;^ EQ-VAS=EQ visual analogue scale.

^*^significant *P*-values (*P* < 0.05).

**Table 3:** Model means of FKSI-15 item scores across all available post-baseline observations (mixed-effects model)

	**Total group**				**EU group**				**US group**				**US *vs* EU**	
**FKSI-15 items**	**SU**	**IFN-*α***	**Diff.**	***P*-value**	**SU**	**IFN-*α***	**Diff.**	***P*-value**	**SU**	**IFN-*α***	**Diff.**	***P*-value**	**Diff. US *vs* diff. EU**	***P*-value**
*Signs and symptoms*														
**I am losing weight**	3.56	3.35	0.21	0.0037^*^	3.53	3.37	0.16	0.0661	3.48	3.35	0.13	0.0661	−0.03	0.7998
**I have had blood in my urine**	3.98	3.98	−0.00	0.9168	3.97	3.98	−0.00	0.9628	3.97	3.98	−0.01	0.4155	−0.01	0.7304
**I have a lack of energy**	2.62	2.21	0.41	<0.0001^*^	2.56	2.26	0.30	0.0428^*^	2.46	2.14	0.32	0.0009^*^	0.01	0.9293
**I feel fatigued**	2.62	2.35	0.27	0.0078^*^	2.57	2.41	0.16	0.2643	2.45	2.19	0.26	0.0074^*^	0.10	0.5366
**I have pain**	3.06	2.92	0.14	0.0639	2.99	2.98	0.01	0.9235	3.07	2.98	0.08	0.3284	0.07	0.5531
**I have bone pain**	3.28	3.13	0.15	0.0819	3.21	3.17	0.04	0.7628	3.37	3.23	0.14	0.0879	0.10	0.5057
**I am bothered by fevers**	3.88	3.59	0.29	<0.0001^*^	3.86	3.63	0.23	<0.0001^*^	3.89	3.66	0.23	<0.0001^*^	−0.00	0.9939
I am bothered by side effects of treatment	2.71	2.66	0.05	0.6656	2.63	2.81	−0.17	0.2186	2.67	2.47	0.21	0.0528	0.38	0.0209^*^
														
*Respiratory symptoms*														
**I have been coughing**	3.48	3.12	0.36	<0.0001^*^	3.38	3.21	0.17	0.0551	3.46	3.27	0.19	0.0118^*^	0.02	0.8518
**I have been short of breath**	3.35	2.98	0.37	<0.0001^*^	3.33	3.07	0.26	0.0066^*^	3.24	3.01	0.23	0.0084^*^	−0.02	0.8302
														
*Quality of life*														
I am able to enjoy life	2.66	2.37	0.29	0.0047^*^	2.58	2.33	0.25	0.1096	2.96	2.64	0.31	0.0001^*^	0.06	0.7226
I am sleeping well	2.69	2.39	0.31	0.0030^*^	2.62	2.53	0.09	0.4740	2.79	2.65	0.14	0.0921	0.06	0.6902
I have a good appetite	2.33	2.04	0.29	0.0344^*^	2.28	1.88	0.40	0.0322^*^	2.58	2.36	0.23	0.0512	−0.18	0.4050
I am able to work	2.30	1.90	0.40	<0.0001^*^	2.21	1.92	0.29	0.019^*^	2.57	2.35	0.22	0.0328^*^	−0.07	0.6267
														
*Emotional symptom*														
I worry that my condition will get worse	2.68	2.55	0.13	0.0969	2.59	2.46	0.13	0.269	2.82	2.63	0.19	0.0241^*^	0.06	0.6405

Abbreviations: SU=sunitinib; IFN-*α*=interferon-alfa; Diff.=sunitinib *vs* IFN-*α*; ^*^Significant *P*-values (*P*<0.05).

FKSI-15=Functional Assessment of Cancer Therapy–Kidney Symptom Index15 item; the nine **bold** items make up the Disease-Related Symptoms subscale (FKSI-DRS).
